# Supplementary material for: Change of deep subduction seismicity after a large megathrust earthquake
Source: Nat Commun. 2024 Jan 2;15:60. doi: 10.1038/s41467-023-43935-3 (PMC10761866; doi:10.1038/s41467-023-43935-3)
Supplement: Supplementary file 1 — Supplementary Information [file 41467_2023_43935_MOESM1_ESM.pdf]

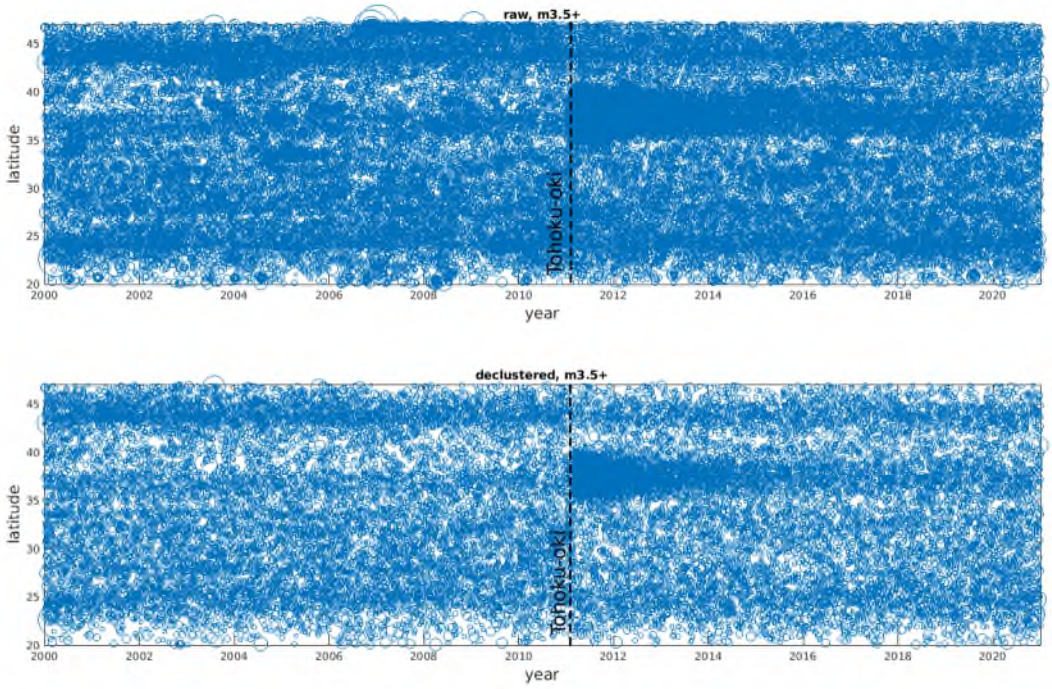

Fig. S1: Comparison of the raw and declustered catalog for all the earthquakes. (top) all magnitude ≥3.5 earthquakes in the JMA catalog with no condition on depth. The circle radius grows with the magnitude. Aftershock sequences are readily visible as clusters initiated by large shocks. (Bottom) same as top graph after declustering. The aftershock sequences are all removed except for the Tohoku-oki earthquake aftershocks where we still see a large number of aftershocks even after declustering for shallow depth earthquakes. Note that in this study, we focus on the earthquakes deeper than 150km (see Fig. S2).

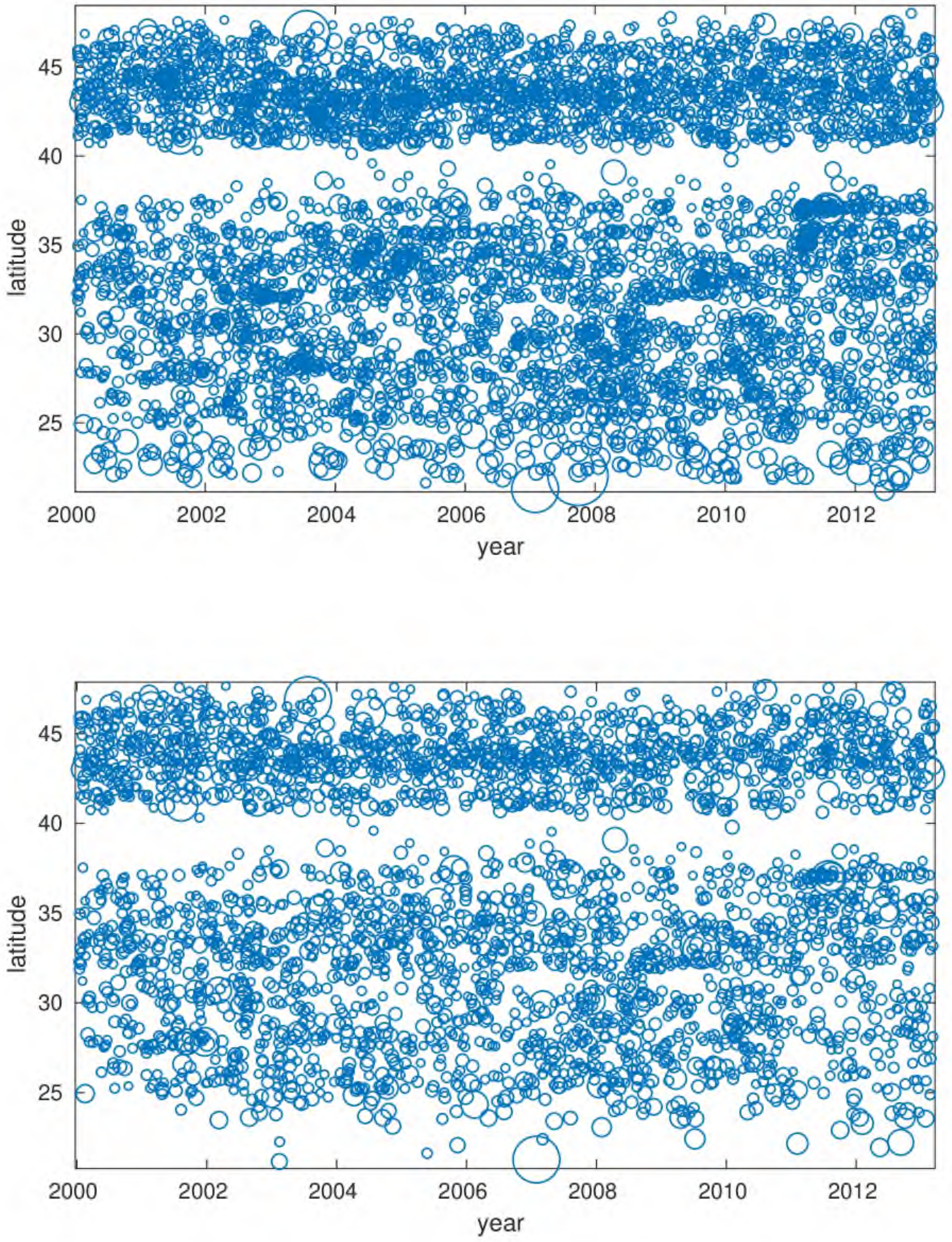

Fig. S2. Comparison of the raw and declustered catalog for deep earthquakes. Magnitude ≥ 3.5 earthquakes, deeper than 150km, before (top) / after (bottom) declustering for the time period used in the statistical study. Note that the latitudes between 37°N and 40°N correspond to the scarse seismicity underneath the Japan Sea.

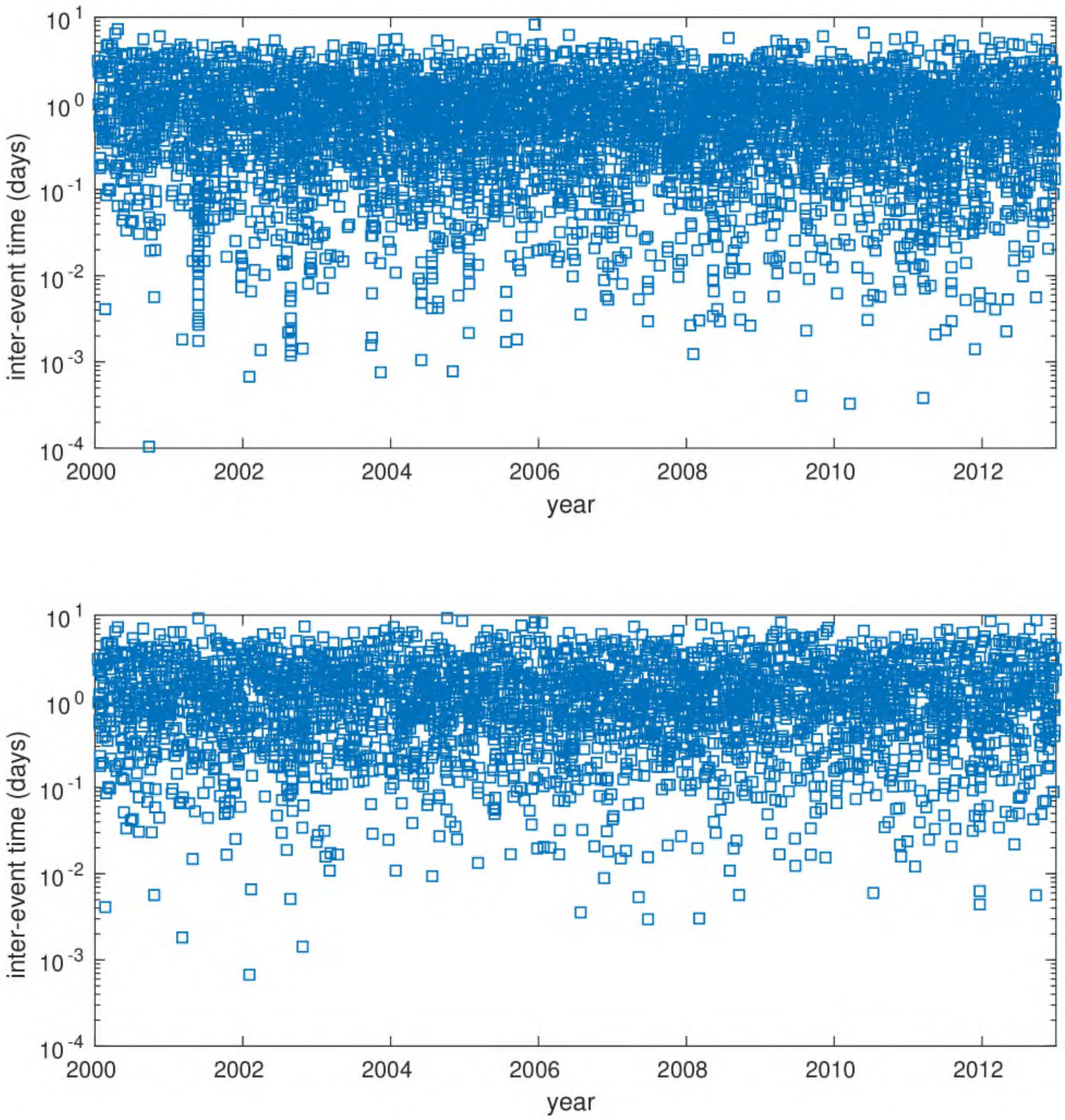

Fig. S3. Inter-event time of two successive earthquakes before and after declustering. Time between two successive earthquakes for the selected deep earthquakes, before (top) and after (bottom) declustering. The vertical 'streaks' corresponding to aftershock sequences (e.g., in 2001,

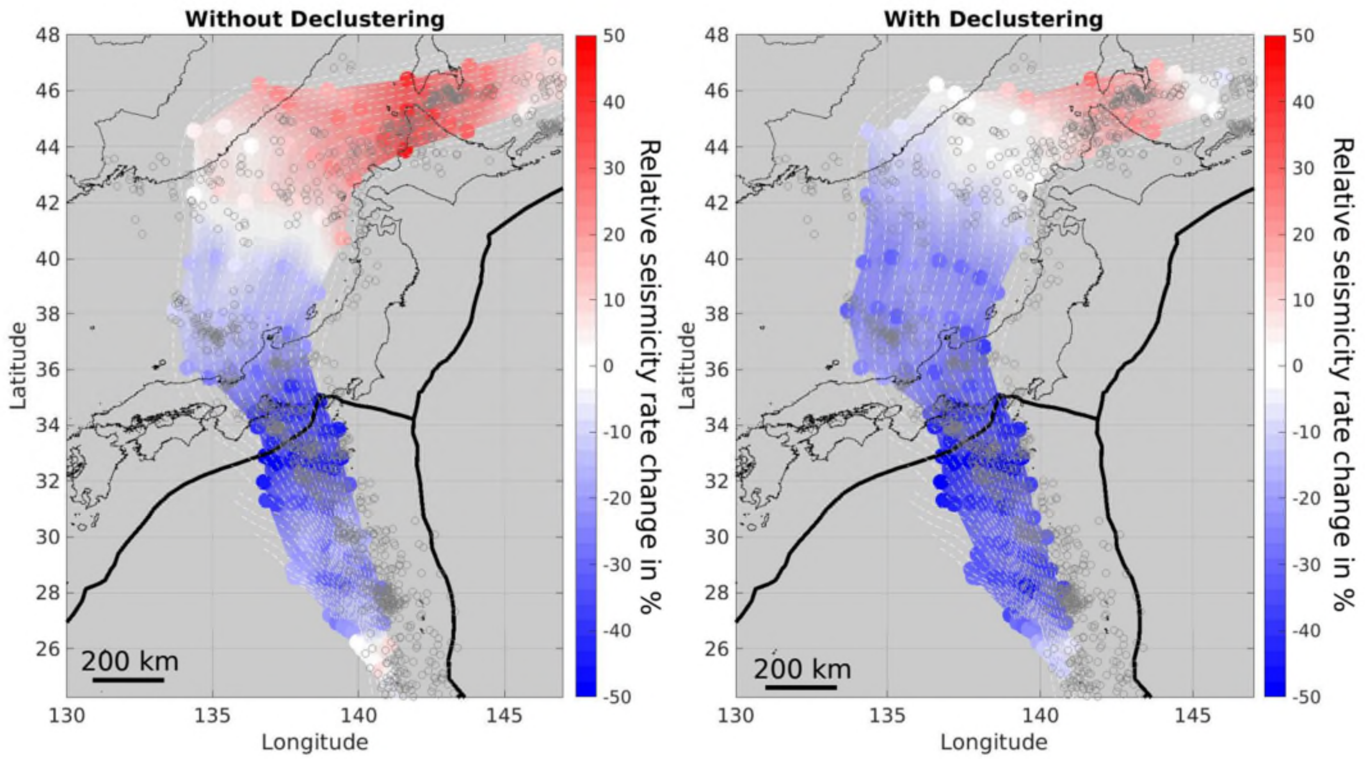

Fig. S4. **Effect of the declustering on the analyzed data.** Comparison of the relative deep seismicity (150-650km) rate change (in %) obtained before (left) and after (right) declustering. We see the dichotomy between North and South is conspicuous. The declustering method allows to unbias the seismicity rate change, especially at latitudes 36 and 40°.

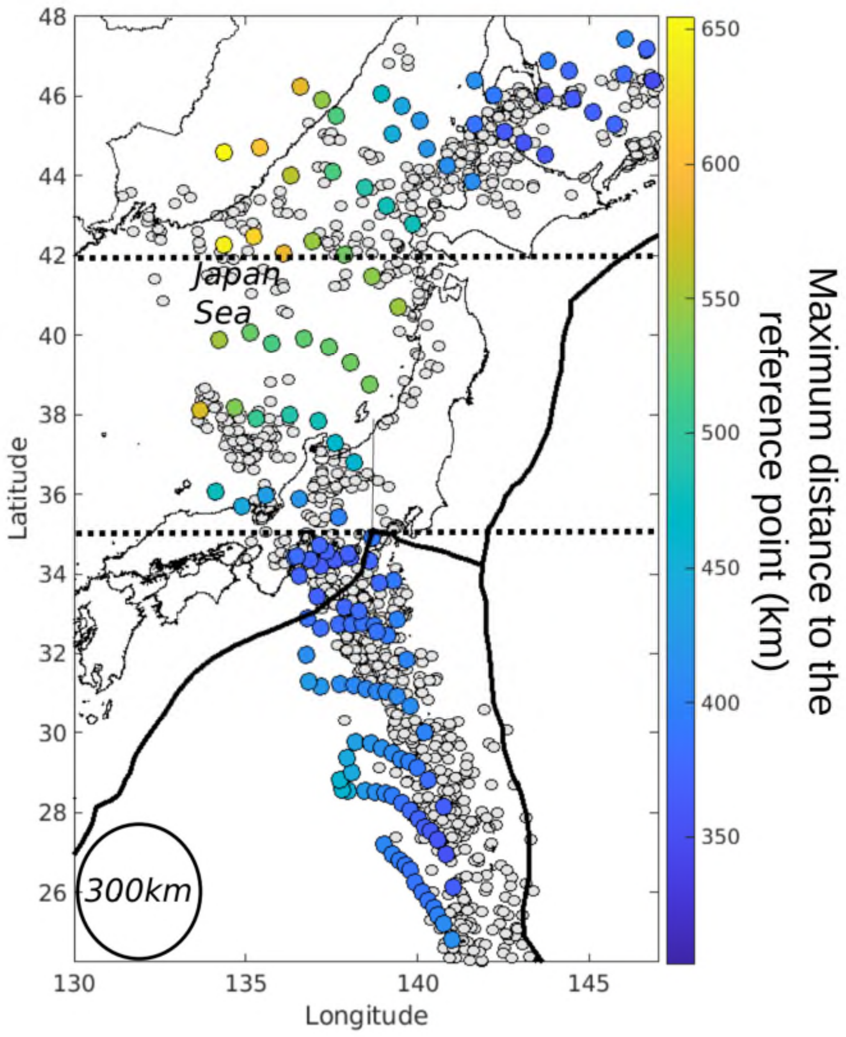

Fig. S5: Maximum distance of between the selected earthquakes to their reference point. Map of Japan showing the location of earthquakes studied in this paper selected with the method described in the main text (gray dots) and location of the reference points color coded with the maximum distance between the point and the farthest earthquake. A circle with a radius of 300km is shown to give a sense of scale.

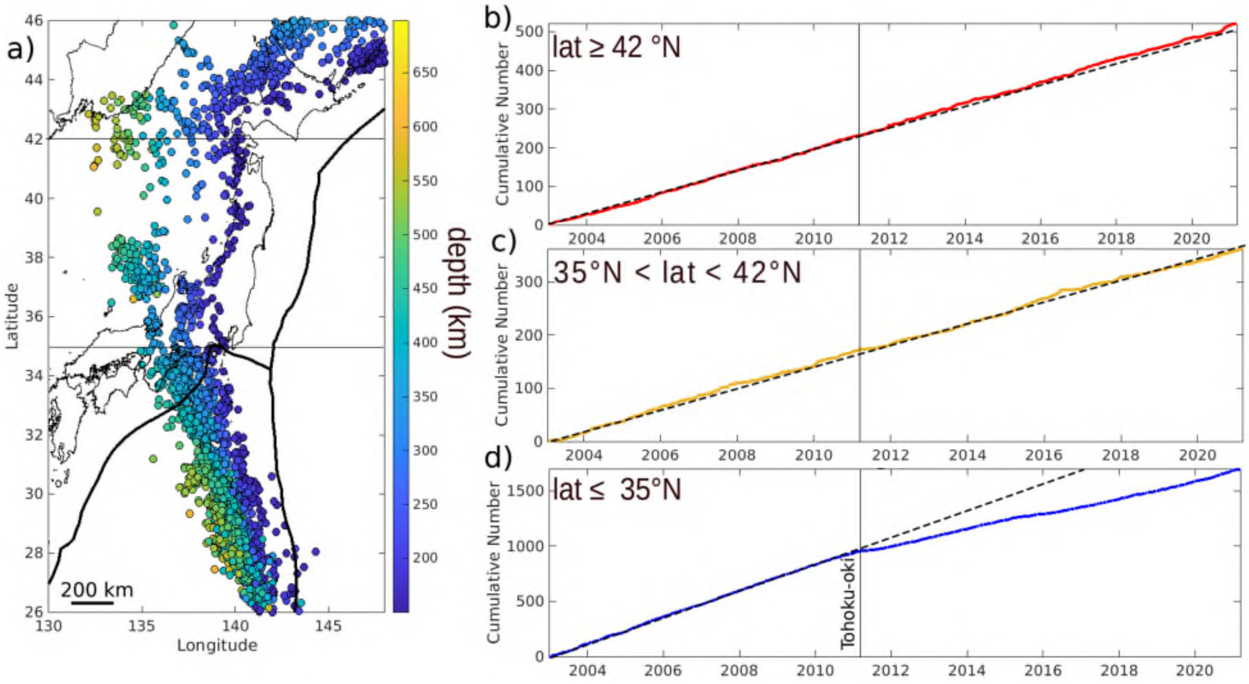

Fig. S6. Location and time series of deep earthquakes in Japan analyzed in this study. a) Location of the earthquakes analyzed in this study, color coded with depth. b) Cumulative number with time of all deep, declustered, earthquakes with magnitude ≥ 3.5 located at latitude > 42°N, c) same as b for 35°N < lat < 42°N earthquakes. d) same as b for lat < 35°N earthquakes. Note that the total number of selected earthquakes is smaller for the Japan Sea (c) than underneath Hokkaido (a) or in the south (b). There is no significant change of seismic rate in this area while there is an increase and decrease in the North and South, respectively.

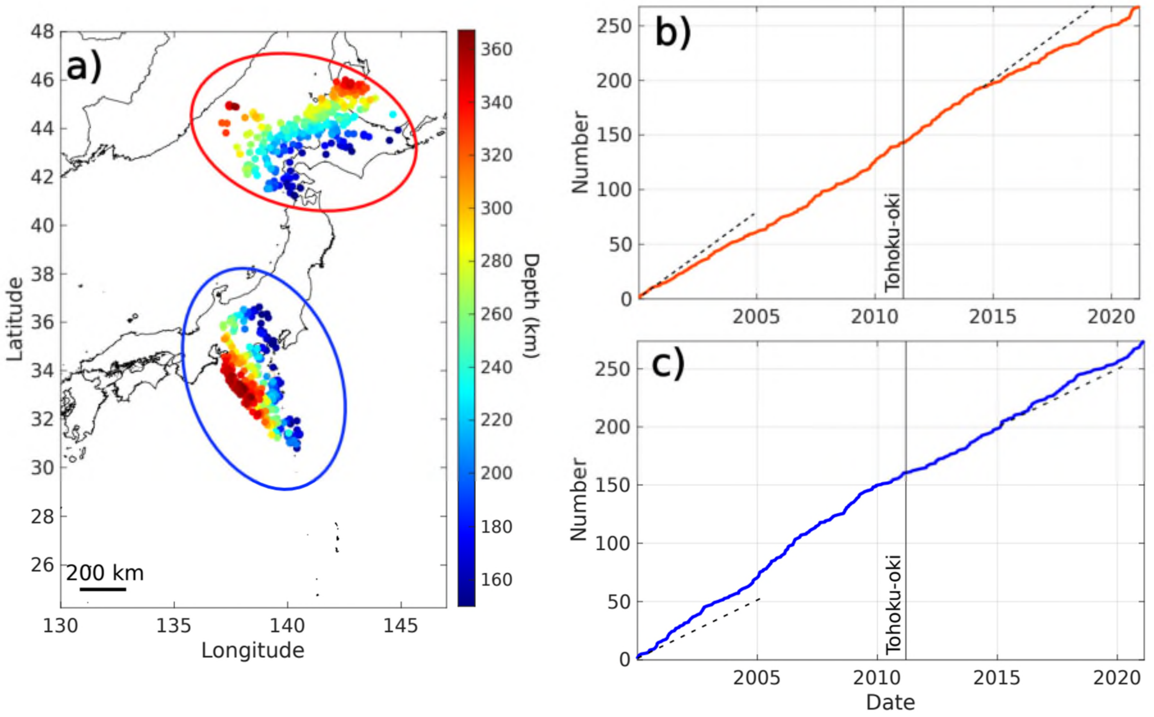

Fig. S7. **Location and time distribution of earthquakes in two zones.** a) Location of the selected earthquakes. b) Cumulative number of earthquakes with time after declustering for 44°N latitude (red) and 34°N latitude (in blue). Note the change of slope at the time of the Tohoku-oki earthquake. In this study, we focus the statistical analysis on the 2011/3/11-2013/3/11 period, shown in the black rectangle. For comparison, the slope of this time period is given by the dotted lines at different times.

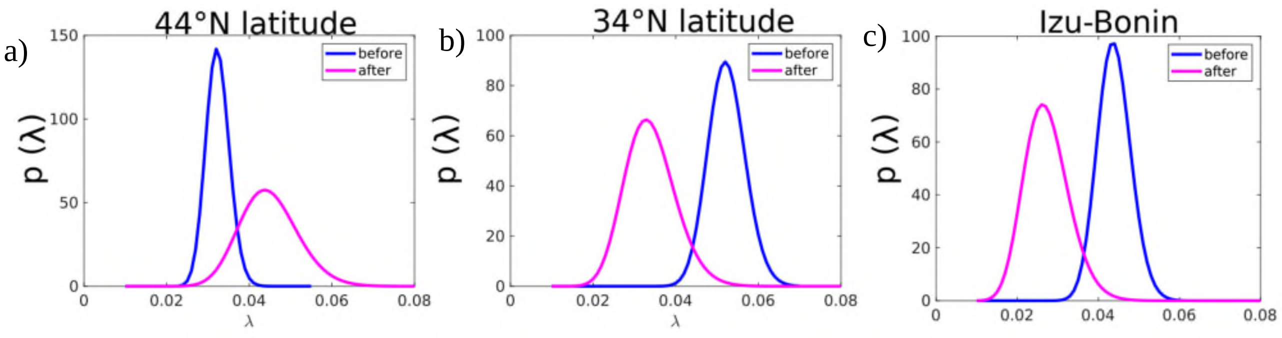

Fig. S8. **Probability density function of the earthquake rate p(λ), in 1/day.** Observed in a) Hokkaido (44°N latitude), b) 34°N latitude and c) Izu-Bonin area : before, i.e. λ_b (2000/3/11 – 2011/3/10, blue) and after, i.e. λ_a (2011/3/11 – 2013/3/11, pink) the Tohoku-oki earthquake. Note that with more earthquakes before the distribution of the λ is sharper before than after the Tohoku-oki earthquake.

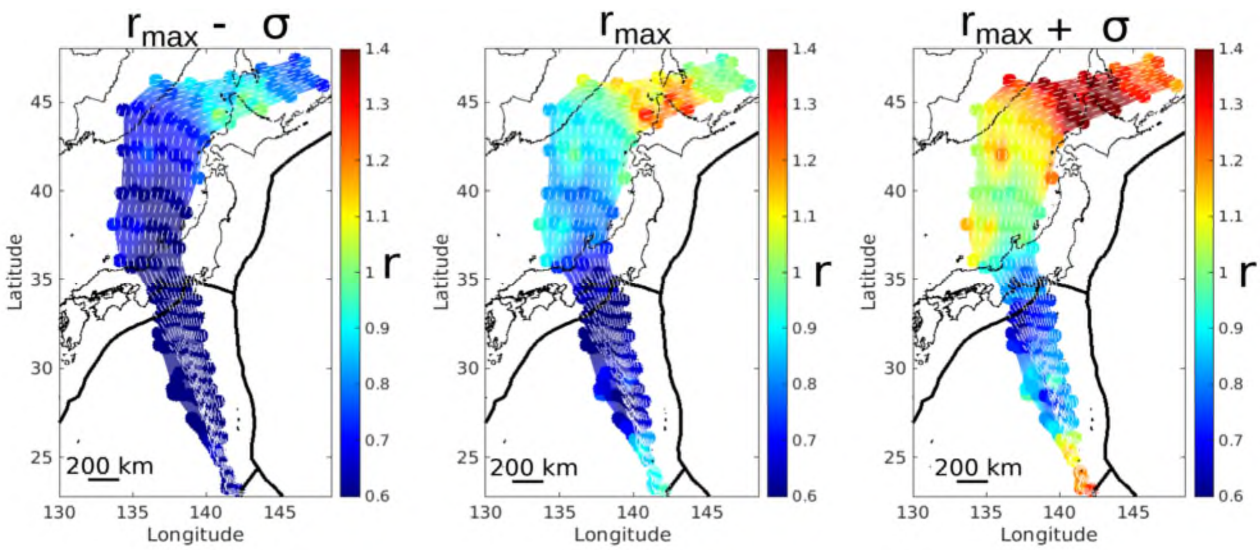

Fig. S9. **Analysis of the uncertainty on the maximum of the pdf of r**. To show the uncertainty on r_{max}, i.e. the r value that maximizes f(r) (middle), we show the r-values r_{max}-σ (left) and r_{max}+σ (right) with the same colorbar for comparison. Even after removing 1σ to the maximum, there is still an increase in the seismicity rate underneath Hokkaido, and still a decrease in seismicity rate at latitude 34° when adding σ. Note that the data in the middle is the same as in Fig. 1 with the difference that the scale is here not in percentage.

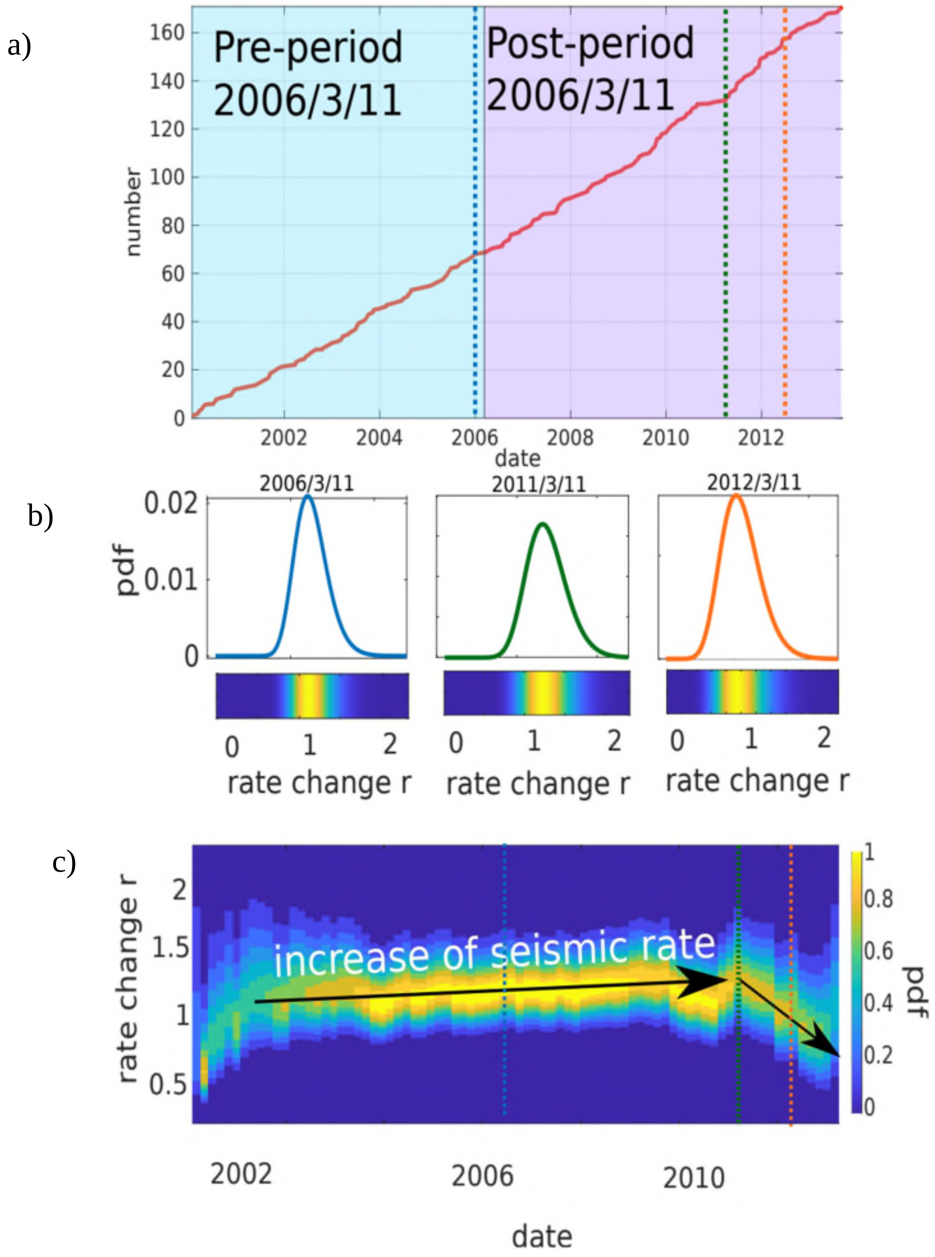

Fig. S10 Computation of the pdf of the seismicity rate change at each time plot for different times of change underneath Hokkaido. a) cumulative number of earthquakes with time, b) pdf of r, c) pdf of r for different times of change. The example shows that in 2006 (blue line), the increase of the seismicity rate due to the Tohoku-oki earthquake is in the post-period (between 2006/3/11 and 2013/3/11) so the r-value that maximizes the pdf (blue line) is slightly above 1. Choosing Tohoku-oki as the time of change, the pdf maximum is obtained for r_{max}=1.4. Choosing a time of change in 2012 gives a r < 1 because a part of the increase is in the pre-period (between Tohoku-oki and 2012). We see that r >1 before Tohoku-oki followed by a significative change of slope at the time of Tohoku-oki when r decreases after the megathrust earthquake.

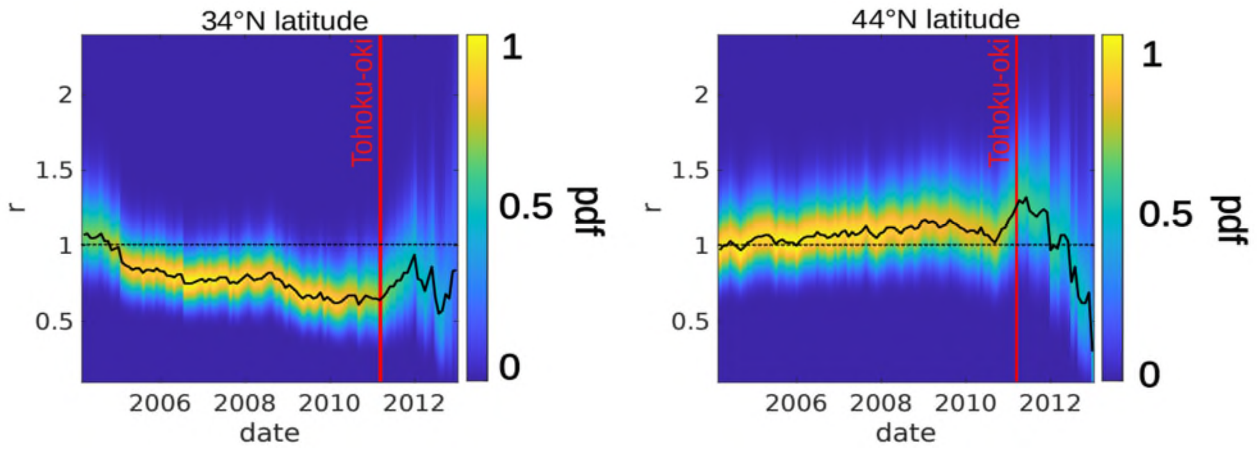

Fig. S11: pdf of the seismicity rate change. We show the pdf computed at each time for 34°N latitude (left) and 44°N latitude (right) with the black line showing r_{max}. A wider pdf means that the level of change is less constrained, as it is the case when one of the 2 periods contains a lower number of events.

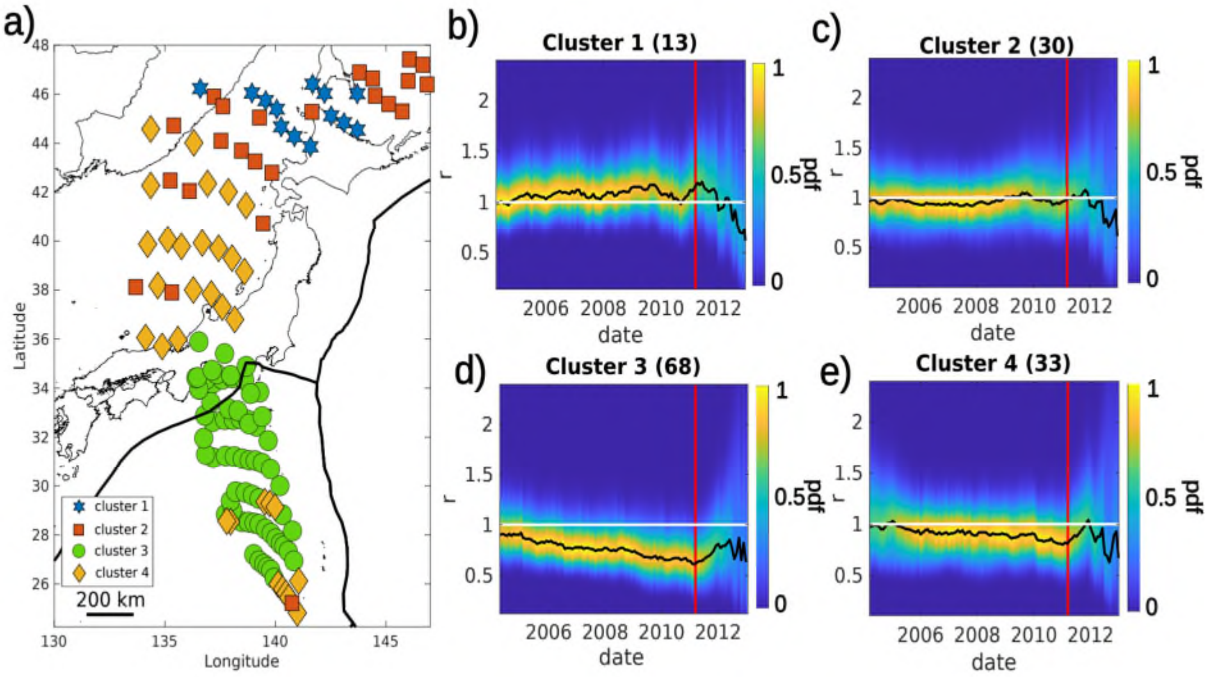

Fig. S12. Location and pdf of the seismicity rate change at each time of the clusters using the k-means clustering on the r_{max} value at the time of the Tohoku-oki earthquake. The number of points present in each cluster is given in brackets. b-e) the black line shows r_{max} and the red line shows the Tohoku-oki earthquake occurrence time. Note that points in the northern and southern tips belong to clusters 2 and 4 which undergo less effect of the Tohoku-oki earthquake indicating the spatial limit of the Tohoku-oki effect.

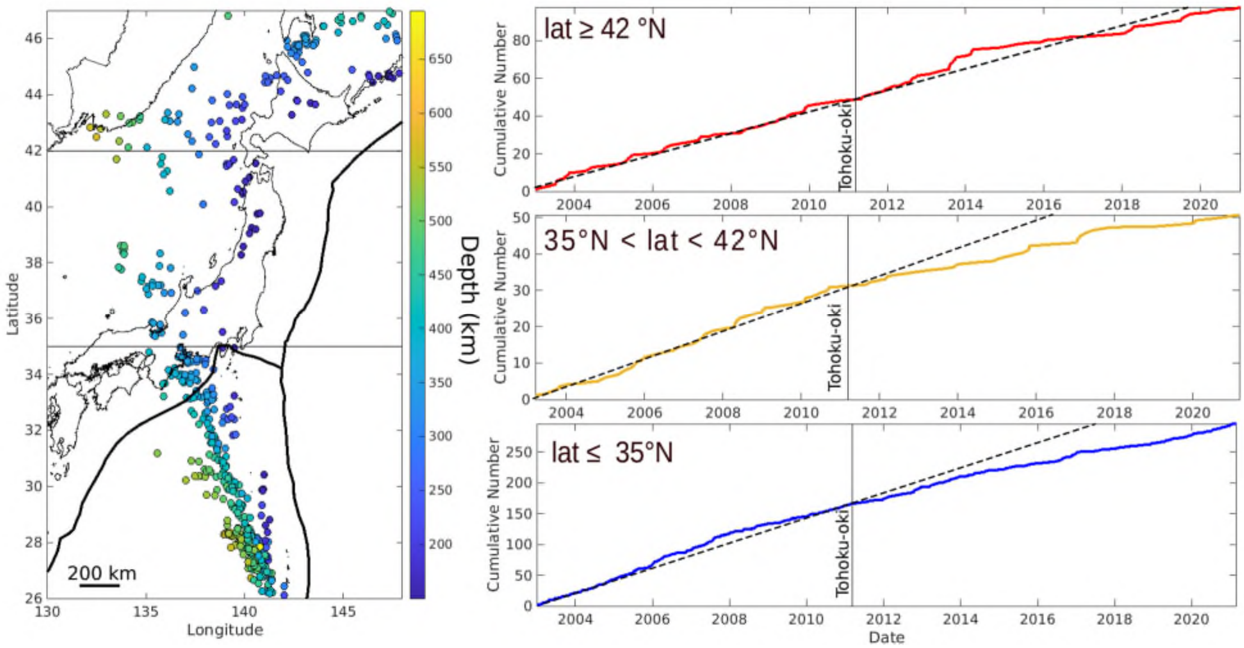

Fig. S13. Same as Figure S6 for declustered earthquakes deeper than 150km and magnitude larger than 4.5.

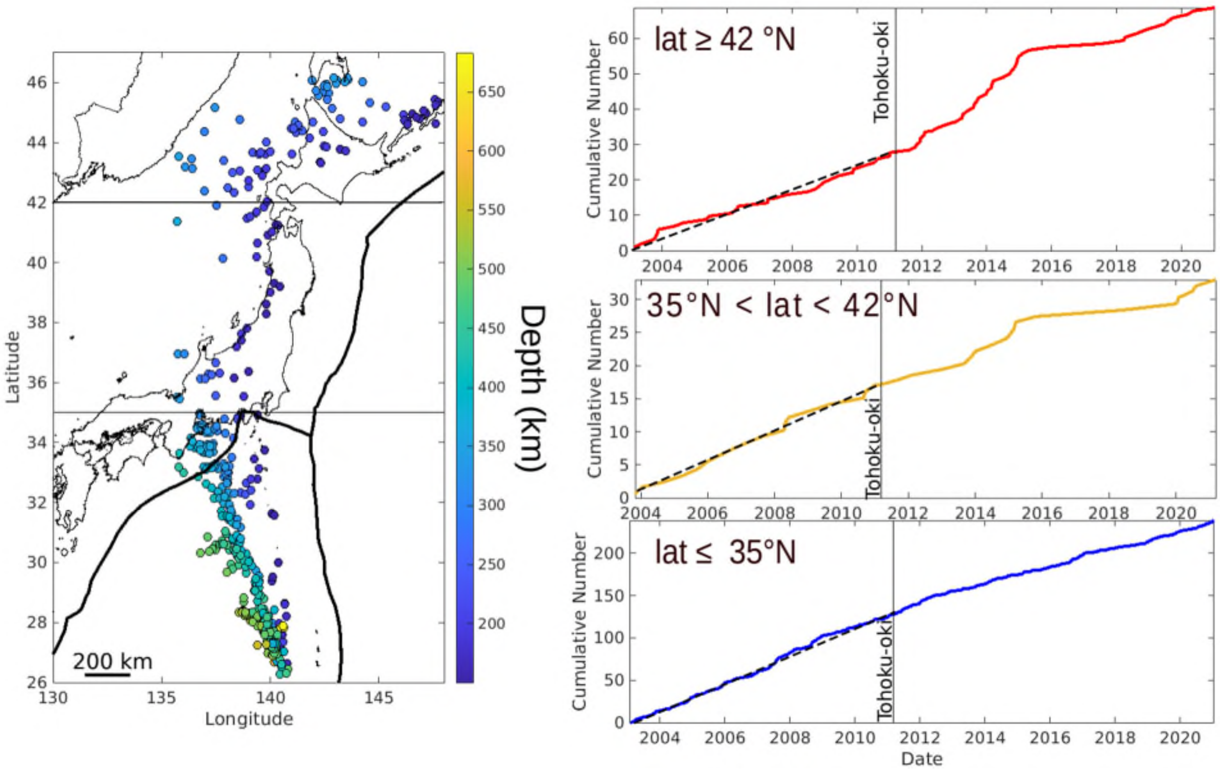

Fig. S14. Same as Figures S6 and S12 using the USGS catalog with earthquakes deeper than 150km and magnitude larger than 4.5.

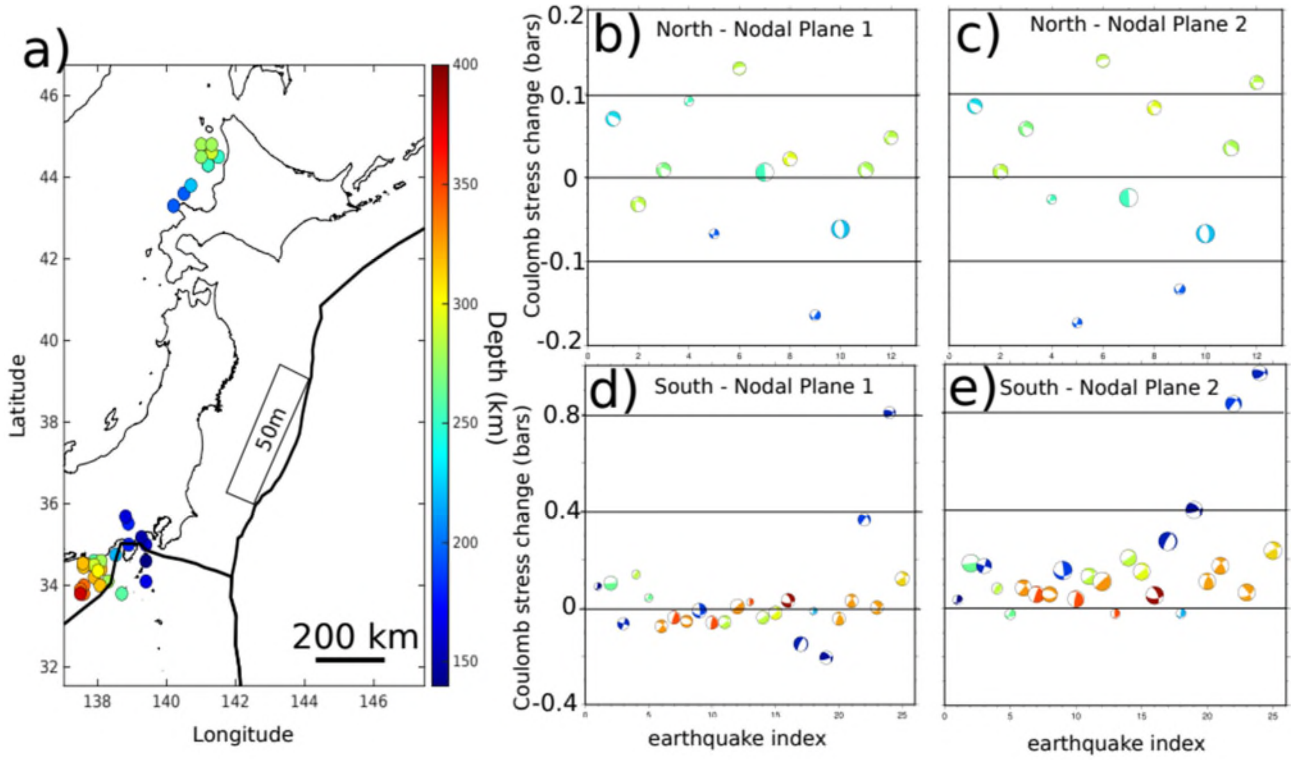

Fig. S15. **Coulomb stress computation of earthquakes underneath Hokkaido and in the South.** a) Location of the earthquakes used to compute the Coulomb stress change color coded with depth. We find only 12 earthquakes close to the points in the North and 25 in the South. We show the 50m of slip dislocation used for the computation; b) Coulomb stress change of the earthquakes located underneath Hokkaido computed using the first nodal plane, we show their focal mechanisms color coded with depth. c) same for the second nodal plane. d) and e) same as b) and c) for the South. This show that, when considering the first nodal plane, the Coulomb stress change is mostly positive for earthquakes underneath Hokkaido (ie promoting slip) except for three southernmost ones. We find mostly negative Coulomb stress change (ie preventing slip) with very diverse focal mechanisms. Two earthquakes that are the northernmost ones present positive Coulomb stress changes.

2010	8	24	13	50	0	44.3	141.2	230	156	36	-50	291	63	-115	0.058	0.03	0.07
2013	1	22	13	29	0	44.5	141.1	280	188	58	-30	295	65	-144	-0.014	-0.046	-0.032
2016	1	11	17	8	1	44.4	141.2	265	178	51	-27	286	69	-138	0.021	-0.031	0.009
2017	1	15	12	13	21	44.3	141.2	251	85	65	151	188	64	28	-0.004	0.237	0.091
2005	9	20	11	59	0	43.6	140.5	195	103	66	177	194	88	24	-1.816	2.862	-0.671
2005	12	11	8	25	0	44.8	141.0	280	75	18	-89	254	72	-90	0.973	0.816	1.299
2009	7	10	18	16	0	44.5	141.5	250	247	10	160	357	87	80	0.038	0.06	0.062
2010	7	19	8	21	0	44.6	141.3	290	176	57	-19	276	75	-145	0.347	-0.328	0.216
2016	8	5	3	30	45	43.3	140.2	195	123	36	179	213	89	54	-2.14	1.329	-1.60943
2017	1	7	9	35	14	43.8	140.7	222	169	34	-96	356	56	-86	-0.605	-0.02	-0.614
2017	9	9	10	12	44	44.5	141.0	276	183	36	-34	301	71	-121	0.136	-0.117	0.089
2018	10	25	10	25	35	44.8	141.3	279	175	46	-8	270	85	-135	0.528	-0.136	0.474

Table 1 : Location and focal mechanisms of the 12 earthquakes closest to the point underneath Hokkaido. Format : year, month, day, hour, minute, second, latitude, longitude, depth (km), strike 1, dip 1, rake 1, strike 2, dip 2, rake 2, shear stress (bar), normal stress (bar), Coulomb stress change (bar) for the first nodal plane.

2002	5	15	10	23	0	34.6	139.4	140	71	35	151	185	74	58	0.134	-0.114	0.088
2003	10	19	5	21	0	34.6	137.9	260	82	88	-99	338	9	-14	0.121	-0.047	0.102
2004	3	20	16	18	0	34.1	139.4	165	19	84	1	289	89	174	0.101	-0.417	-0.066
2004	9	4	6	12	0	34.1	138.3	280	37	30	95	211	60	87	0.135	0.7	0.137
2004	11	3	20	13	0	33.8	138.7	260	93	48	154	201	71	46	0.057	-0.038	0.041
2005	5	28	20	55	0	34.5	137.7	320	40	88	-150	308	60	-2	-0.011	-0.163	-0.077
2006	3	4	18	38	0	33.8	137.6	340	201	82	106	317	17	27	0.016	-0.138	-0.040
2006	4	28	22	16	0	34.4	138.1	320	86	46	-111	294	48	-70	-0.037	-0.047	-0.056
2007	1	15	18	18	0	35	138.9	180	93	39	-169	354	83	-52	0.021	-0.082	-0.012
2007	7	28	8	55	0	34	137.6	340	191	80	104	316	17	37	-0.011	-0.125	-0.061
2008	2	22	8	24	0	34.5	138	280	52	81	-131	311	41	-14	0.013	-0.18	-0.059
2008	3	5	16	2	0	34.2	137.9	320	228	84	98	353	10	36	0.065	-0.148	0.5
2009	6	17	16	24	0	33.90	137.5	340	53	21	142	179	78	73	0.014	0.022	0.023
2010	8	29	4	27	0	34.60	138.1	280	55	87	-104	311	14	-140	0.035	-0.186	-0.040
2011	1	22	2	48	0	34.50	137.9	290	51	90	-120	321	30	0	0.048	-0.173	-0.021
2013	9	23	8	7	0	33.80	137.5	380	87	58	-148	338	63	-37	0.044	-0.033	0.03
2014	5	4	20	18	0	35	139.4	160	208	75	-80	354	18	-123	0.043	-0.484	-0.15
2015	3	13	15	12	48	34.7	138.5	218	85	30	170	184	85	60	-0.7	-0.018	-0.014
2016	1	18	0	56	48	35.2	139.27	150	72	71	50	321	43	152	-0.057	-0.372	-0.206
2017	7	18	7	47	1	34.5	137.6	316	205	70	127	319	41	31	0.010	-0.136	-0.045
2019	4	29	5	24	0	34.5	137.6	318	226	74	155	323	66	18	0.082	-0.124	0.028
2020	2	2	6	28	8	35.5	138.9	180	218	88	-49	310	41	-178	0.449	-0.212	0.364
2020	6	21	4	55	19	33.9	138.0	317	60	57	-167	323	79	-34	0.041	-0.102	0.1
2020	7	24	19	51	33	35.7	138.8	162	46	51	37	291	62	135	0.709	0.242	0.805
2020	10	6	23	2	6	34.4	138.0	302	242	83	144	337	54	9	0.164	-0.110	0.120

Table 2: Location and focal mechanisms of the 12 earthquakes closest to the point underneath 34°N latitude. Format : year, month, day, hour, minute, second, latitude, longitude, depth (km), strike 1, dip 1, rake 1, strike 2, dip 2, rake 2, shear stress (bar), normal stress (bar), Coulomb stress change (bar) for the first nodal plane.
